# *In vitro* performances of novel co-spray-dried azithromycin/rifampicin microparticles for *Rhodococcus equi* disease treatment

**DOI:** 10.1038/s41598-018-30715-z

**Published:** 2018-08-14

**Authors:** Elisa Rampacci, Maria Luisa Marenzoni, Elisabetta Chiaradia, Fabrizio Passamonti, Maurizio Ricci, Marco Pepe, Mauro Coletti, Stefano Giovagnoli

**Affiliations:** 1 Department of Veterinary Medicine, Centro di Studio del Cavallo Sportivo, University of Perugia, Via San Costanzo 4, Perugia, 06126 Italy; 2 Department of Pharmaceutical Sciences, University of Perugia, Via del Liceo 1, Perugia, 06123 Italy

## Abstract

This work was aimed at providing clues on the *in vitro* performances of novel azithromycin/rifampicin combinations, in the form of co-spray-dried microparticles (AZM/RIF MP), against *Rhodococcus equi*, an animal and emerging human pathogen found responsible for worrying zoonosis. Various AZM/RIF combinations were spray-dried and characterized for their morphology and size. Susceptibility studies included determination of MIC, MBC, Fractional Inhibitory/Bactericidal Concentration Indexes and intracellular activity in *R*. *equi*-infected THP-1 cells. Cytotoxicity was tested on BEAS-2B cells through MTT assay and combination index assessment for drug interaction. Spray-dried MP were collapsed and 3–10 times smaller than commercial powders. Drug combinations showed an enhancement of *in vitro* antibacterial activity with a remarkable synergistic bactericidal effect. Azithromycin MP and AZM/RIF MP 2:1 led to a CFU reduction of >90% up to 4 days after treatment at all tested concentrations (p = 0.001) but AZM/RIF MP 2:1 were at least four-fold more potent than AZM MP alone. IC_50_ values of >100 mg/L supported low cytotoxicity of drug combinations and the combination index suggested an antagonistic toxic effect. Co-spray-drying enhanced powder dispersibility and solubility, which may improve bioavailability as well as provide administration alternatives. The novel AZM/RIF MP combinations could result a valid platform to develop new treatment strategies against *R*. *equi* infections in animals and humans.

## Introduction

The worldwide need for effective antimicrobial regimens has encouraged an ever-growing research effort towards a combinatorial approach for the treatment of specific conditions affecting both animals and humans. Such an approach has become essential for current therapies to treat increasing drug resistant infections, as in the case of mycobacteria-like diseases in human and veterinary medicine. Among these, *Rhodococcus equi* disease is an important equine pathology causing chronic pneumonia in foals with high morbidity and mortality rates^1^. This facultative intracellular bacterium is widely distributed in nature and considered one of the leading opportunistic and zoonotic pathogens in humans^2–4^. Human rhodococcosis, characterized by pulmonary and/or extrapulmonary forms, has increased globally as a result of HIV epidemic and prolonged immunosuppressive therapies for cancer and organ transplant^5^. Such a disease is frequently misread as *Mycobacterium tuberculosis* infection because of similar clinical features and positive Ziehl-Neelsen sputum specimen^6,7^. Additionally, genome sequencing of *R*. *equi* ATCC 33701 revealed a close gene homology with *M*. *tuberculosis*^8^.

The association of a macrolide with rifampicin (RIF) is the current therapy regimen in foals with clinically apparent rhodococcosis^1^ and deeply recommended for treating human pathology^9^. However, *in vitro* and *in vivo* data are not sufficient to support a consensual optimal treatment for rhodococcosis. RIF has a high Mutant Prevention Concentration (MPC) against *R*. *equi*. Combining RIF with a macrolide such as azithromycin (AZM), the MPC against *R*. *equi* decreases^10^. RIF appears to be able to interrupt the immunogenic mechanism^11^. This effect combined with antimicrobial drugs that interfere with protein synthesis, such as AZM, may allow the eradication of intracellular organisms^12^. Therefore, in this work, we investigated the *in vitro* performance of AZM/RIF combinations against *R*. *equi*. In particular, we focused our attention on (1) defining the potential synergistic effect of spray-dried AZM/RIF combinations against virulent *R*. *equi* by comparing extracellular and intracellular activities, (2) identifying and characterizing the most effective drug combination and (3) assessing cytotoxicity of AZM/RIF combinations.

In order to enhance their action, AZM/RIF combinations were formulated as dry powders by a spray-drying process. In this way, small and relatively homogeneous microparticles (MP) can be formed with precise drug molar ratios and a potentially higher dispersibility as a result of a reduced size, increased surface area as well as drug amorphous state. In particular, the delivery of antimicrobial combinations in the form of dry MP, where multiple drugs are combined together in a single particle, has never been explored for *R*. *equi* infections in humans or animals. Therefore, such particulate systems can provide multiple advantages including the possibility of oral or pulmonary administration^13,14^. Orally administered, these MP may improve dissolution thus potentially enhancing bioavailability^15^. If inhaled, they may grant a more homogeneous deposition of the selected drug ratio in the airways alongside the well-known advantages of lung delivery, which include reduced dosages as well as gastro-intestinal and systemic side-effects^16^.

Overall, the investigation of *in vitro* performances of the proposed spray-dried AZM/RIF combinations may represent a platform for the future development of a new combination treatment of *R*. *equi* infections.

## Materials and Methods

### Preparation of spray-dried microparticles

Three binary AZM/RIF combinations, 1:1, 2:1 and 1:2, were transformed into MP formulations by spray-drying technology. AZM MP and RIF MP spray-dried powders were prepared independently for comparison. The method is a one step process that allow to obtain dry powders with improved morphological and dimensional characteristics. The microparticles were produced by a Mini Spray-Dryer Model B-290 (Büchi, Milan, Italy) starting from excipient-free drug solutions at a final concentration of 2% w/v in acetonitrile. The spray-drying parameters were set up as follows: inlet temperature 75 °C, air flow rate 357 L/h, feed rate 2.5 mL/min and aspirator rate 20 m^3^/h.

### Characterization of MP dry powders

The particle size was determined through SPOS (single particle optical sensing) system using an Accusizer C770 (PSS Inc., Santa Barbara, CA, USA) after having suspended dry powders in distilled water. Morphological characterization was performed by scanning electron microscopy (SEM) employing a FEG LEO 1525 high resolution microscope equipped with a GEMINI column (ZEISS, Jena, Germany). Dry-powders were deposited onto an aluminum stub covered with a double sided adhesive carbon disc and coated with chromium at 120 mA for 30 s prior to imaging. Size was expressed as number-weighed mean diameter (NMD) and volume-weighed mean diameter (VMD). Broadness of size distribution was determined as population *span* as reported in Equation 1.

1 $$span=\frac{{d}_{{90}}-{d}_{{10}}}{{d}_{{50}}}$$

where *d*_*10*_, *d*_*50*_, and *d*_*90*_ are the particle diameters at 10, 50 and 90%, respectively, of the population distribution.

Drug content (%DC) in each AZM/RIF formulation was determined by a HPLC method using an HP1050 HPLC system with UV-vis detector (Hewlett-Packard, Waldbronn, Germany). The conditions were as follows: Gracesmart^TM^ C18, 5 µm, 250 × 4.6 mm column conditioned at 25 °C, mobile phase 50:50 acetonitrile/10 mM pH 7.2 phosphate buffer, 1 mL/min flowrate in isocratic mode. Calibration for RIF and AZM was performed in the 0.1–0.55 mg/mL (r^2^ = 0.9997) and 1–4 mg/mL (r^2^ = 0.9994), respectively, λ_max_ = 210 nm.

Drug content was expressed as:

2 $$\, \% DC=\frac{amount\,of\,drug\,measured}{total\,mass\,of\,MP}\ast 100$$

Briefly, an exactly weighed amount of MP was dissolved in acetonitrile and upon proper dilution was submitted to spectrophotometric analysis. All measurements were performed in triplicate and results expressed as mean ± S.D.

Physical state of powders was assessed by thermal analysis using a Mettler Toledo DSC 821 differential scanning calorimeter (Mettler Toledo, Switzerland) equipped with a liquid nitrogen cooling system. The system was calibrated using an indium standard. MP were compared with drug commercial powders. Samples were weighed and hermetically sealed in pin holed lid 40 μL aluminum pans. Heating was performed from 25 to 200 °C, at a 10 °C/min rate. DSC data were treated with STARe software.

Residual solvent in dry powders was determined by thermogravimetric (TGA) analysis using a Netzsch STA 449 C apparatus, in air flow and heating rate of 10 °C/min in the 25–150 °C temperature range.

Dispersibility of the obtained powders was assessed by measuring the scattering signals of dispersed powders by photocorrelation spectroscopy. Briefly, suspensions of drug commercial powders and spray-dried MP were prepared at the same concentration of the stock solutions employed for cell studies in ultrapure water at 20 °C. Analyses were performed by a Nicomp 380 ZLS photocorrelator (PSS, Santa Barbara, California USA) equipped with a 35 mW He/Ne laser (λ = 654 nm) and an Avalanche photodiode detector.

Solubility of RIF and AZM dry powders and their combinations was determined, at 20 °C, in 10 mM phosphate buffer pH 7.2 by using the HPLC method described above. Briefly, exactly weighed amounts of MP and commercial powders were suspended in the medium and left to equilibrate for 10 min. The samples were then centrifuged at 2500 rpm for 5 min (model) and aliquots were opportunely diluted in acetonitrile and submitted to HPLC analysis. All measurements were performed in triplicate.

### Susceptibility studies and synergy evaluation

The lowest concentration of antimicrobial able to inhibit the visible growth of virulent model *R*. *equi* ATCC 33701 (MIC) was established performing the broth microdilution procedure. A 1:1000 dilution was prepared in Cation-Adjusted Mueller-Hinton Broth starting from a 0.5 McFarland standard bacterial suspension, confirmed using spectrophotometric quantification at 600 nm wavelength, to obtain a final concentration of 1.5 × 10^5^ CFU/mL. Each well of 96-well microplates was inoculated with 100 µL of bacterial suspension. The recommended inoculum and the purity were verified by bacterial culture onto 5% defibrinated sheep blood agar and count of diluted aliquots. The susceptibility testing was realized for AZM/RIF MP 1:1, AZM/RIF MP 2:1, AZM/RIF MP 1:2, AZM MP and RIF MP ranged between 0.00095–128 mg/L/component in serial two-fold dilutions in Cation-Adjusted Mueller-Hinton Broth. Positive and negative control wells were tested on each plate then incubated at 37 °C. The standard reading was located at 24 h^17^. The assay was validated if the visible growth in positive controls was retained acceptable. Experiments were performed in triplicates of four independent experiments.

To determine the minimum bactericidal concentration (MBC) the subculture of drug dilutions showing bacterial inhibition was carried out onto antibiotic-free blood agar plates^18^. The plates were read at 18, 24-hour intervals up to 5 days to ensure adequate growth^19^.

The Fractional Inhibitory Concentration Index (FICI) for AZM/RIF combinations was used as predictor of synergy:

3 $${\rm{\Sigma }}\mathrm{FICI}=\frac{MIC\,AZM\,in\,combination}{MIC\,AZM\,alone}+\frac{MIC\,RIF\,in\,combination}{MIC\,RIF\,alone}$$

The Fractional Bactericidal Concentration Index (FBCI) was similarly determined by substituting the MIC with the MBC values. The results of FICI and FBCI calculation were interpreted as follows: value ≤ 0.5 defines synergistic interactions, 0.5 < FICI ≤ 1 partial synergy, 1 < FICI ≤ 4 indifference, FICI > 4 antagonism^20^.

### Assessment of the intracellular activity in monocyte-derived macrophages

The human cell line THP-1 (ATCC TIB-202) derived from an acute monocytic leukemia was grown as cell suspension to a density of 0.8–1 × 10^6^ cells/mL in Roswell Park Memorial Institute-1640 medium supplemented with 10% (v/v) fetal calf serum (FCS) at 37 °C with 5% CO_2_ in a humidified incubator^21^. Differentiation of monocytes was induced by 24 h exposure to 20 ng/mL of phorbol 12-myristate 13-acetate in 24-well plates to obtain monolayers expressing macrophage-like phenotype. The monolayers were washed twice with sterile phosphate-buffered saline to remove non-adherent cells. Subsequently, THP-1 cells were infected in triplicate with 100 µL of *R*. *equi* ATCC 33701 suspension equivalent to 1.5 × 10^8^ rhodococci/mL for inducing a multiplicity of infection of 10–20 bacteria/cell (MOI 10–20)^22^ and incubated for 1 h to allow phagocytosis. Two washes with sterile phosphate-buffered saline were performed prior to treat cells with 1 mL/well of each antimicrobial solution to remove unphagocyted bacteria. Treatment groups included triplicates of AZM/RIF MP 1:1, AZM/RIF MP 2:1, AZM/RIF MP 1:2, AZM MP and RIF MP at MBC, MIC and MIC/2, infected macrophages in Roswell Park Memorial Institute-1640 medium supplemented with 10% (v/v) FCS and non-infected cell controls in culture medium. Considered the slow cultivation of *R*. *equi* and our interest in observing a potential long-term activity of antibiotics, post-treatment follow-up time intervals of 2, 4, 6 and 8 days were chosen to evaluate bacterial long-term growth curves in treated and control. Adherent cells in each well were treated with 100 µL of 0.25% sodium dodecyl sulfate for 30 min at 37 °C, neutralized by 100 µL of 1% bovine serum albumin. Finally, 30 µL of lysates and their 10^−2^, 10^−3^, 10^−4^,10^−5^ dilutions were inoculated in triplicate onto 5% sheep blood agar plates. The plates were incubated for 18–24 h at 37 °C with 5% CO_2_ in a humidified incubator and the results were expressed as percentage reduction of CFU ± S.D. as compared to the growth of bacteria in untreated controls.

### MTT assay for cytotoxicity testing and Combination index

Evaluation of cellular viability after treatment with AZM MP, RIF MP, AZM/RIF MP 1:1, 2:1 and 1:2 was performed by MTT colorimetric assay on human bronchial epithelial cells ATCC BEAS-2B CRL-9609. This cell line is predicted to provide a reliable witness based on the role played in metabolic process of drugs^23^ and physicochemical factors that seem to favor the accumulation of drugs in question, particularly AZM, in acid environments such as airways under respiratory tract infection^24–26^ and higher concentration than other anatomical districts in healthy subjects^27,28^. The cell line was maintained in Dulbecco’s Modified Eagle Medium supplemented with 10% v/v FCS, 1% glutamine and 1% penicillin-streptomycin at 37 °C in a 5% CO_2_ humidified incubator. Briefly, 5 × 10^3^ cells were seeded in 96-well plates then incubated in humidified chamber at 37 °C with 5% CO_2_ to allow cellular adherence. After 24 h, the medium was replaced with 100 µL of each antimicrobial dilution over the dose range of 0.25–200 mg/L. Negative control, treated with growth medium, were included in each plate as well as positive control consisting of 2 mM of H_2_O_2_. The assay was performed in triplicate and in four independent experiments. After 24 h-treatment time, 10 µL of MTT solution were added to each well containing 90 µL of fresh medium and the plates were incubated for 3 h. Following incubation, the supernatant was removed carefully, the formazan crystals were dissolved in 200 µL of dimethyl sulfoxide and the solution absorbance was measured spectrophotometrically at λ 570 nm. The cellular viability was calculated as ratio between mean absorbance of the sample and mean absorbance of the untreated control and expressed as percentage. Dose-response curves were plotted to determine the half maximal inhibitory concentration (IC_50_) using Graph Pad Prism software 5.00 for Windows. The toxicity of pharmaceutical preparations was categorized as very high if IC_50_ was <1 mg/L, high toxicity 1 < IC_50_ < 10 mg/L, moderate toxicity 10 < IC_50_ < 100 mg/L, low toxicity in case of IC_50_ value > 100 mg/L^29^. Combination index (CI) was used as measure of the combination effect based on IC_50_ of individual drugs and combination treatment^30^.

4 $$\,CI=\frac{(D)\,comAZM}{(D)AZM}+\frac{(D)\,comRIF}{(D)RIF}$$

in which (D)comAZM and (D)comRIF are the IC_50_ in combination for AZM and RIF and (D)AZM or (D)RIF are the IC_50_ of single drugs. The resulting CI allowed to determine whether the cytotoxic effect of the drug combination was additive (CI = 1), synergistic (CI < 1), or antagonist (CI > 1).

### Statistical analysis

The results of the intracellular activity assay were statistically described through ANOVA with post-hoc Tukey HSD and Bonferroni, Kruskal-Wallis H test and Mann-Whitney U-test, as most appropriate. Friedman and Wilcoxon Signed Ranks were carried out to compare readings at different incubation times for each tested concentration in all pairwise comparisons. Data obtained from the MTT assay were analyzed applying one-way and two-way ANOVA with post-hoc Tukey HSD and Bonferroni to determine statistical differences within and among groups. Significance level was predetermined at p ≤ 0.05 for all statistical tests.

## Results

### Characterization of microparticle formulations

**Figure 1:**
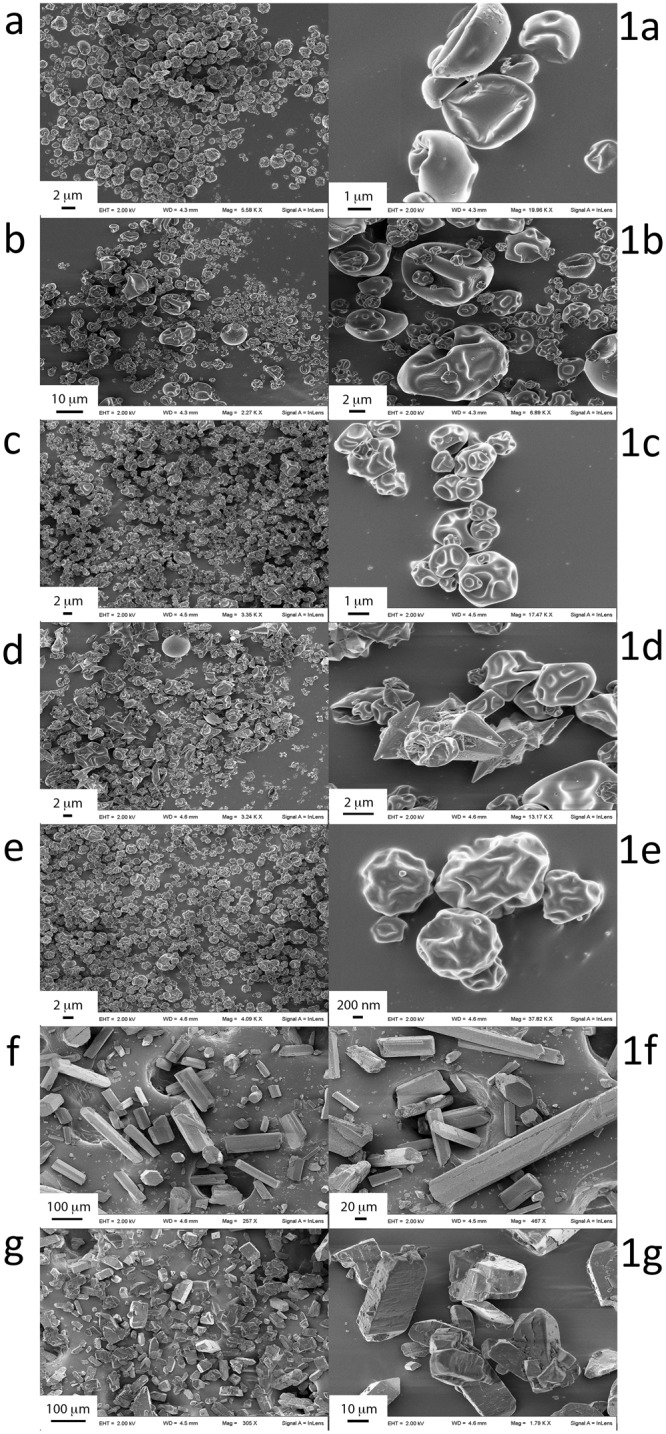
Morphological analysis of the obtained spray-dried powders of AZM and RIF and their combinations in comparison with the commercial powders. AZM/RIF MP 1:2 (**a**,1a), AZM/RIF MP 2:1 (**b**,1b), AZM/RIF MP 1:1 (**c**,1c), RIF MP (**d**,1d), AZM MP (**e**,1e), commercial RIF powder (**f**,1f), commercial AZM powder (**g**,1g). Measurements were performed at 2 kV and images are reported between 0.4–38 kX magnifications.

**Figure 2:**
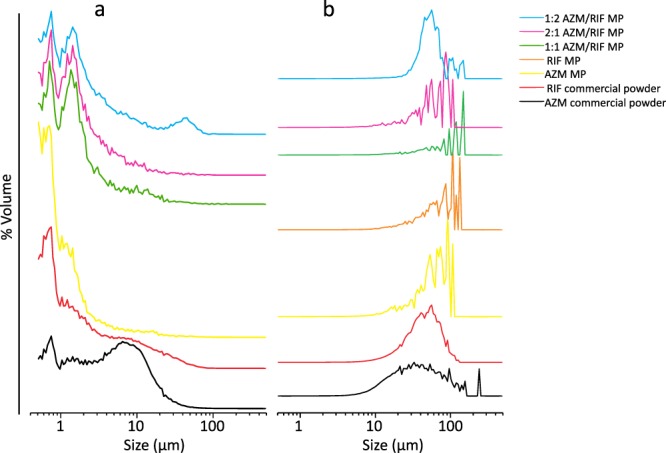
Size distributions of the obtained spray-dried MP in comparison to commercial powders. Number-weighed (**a**) and volume-weighed (**b**) distributions are displayed.

Spray-dried microparticles showed drastic changes compared to commercial drug powders (Fig. 1). AZM and RIF MP and their combinations all assumed a collapsed shape (Fig. 1a–e), typical of spray-dried particles obtained at high buckling conditions, whereas the commercial powders were coarse and irregularly shaped as large rods (Fig. 1f,g). Since acetonitrile was employed as solvent, a fast droplet drying process is expected to produce buckled particles characterized by an empty core. Changing AZM/RIF ratio seemingly did not influence particle morphology. On the other hand, dimensional properties of the MP slightly changed according to the drug and drug ratios employed. In this regard, NMD values were generally lower for AZM as well as in AZM-rich combinations (Table 1). In fact, NMD was the lowest for AZM MP and it increased from 2.4 to 4.8 µm as AZM/RIF ratio decreased. The commercial powders were from 3–10 times larger. *Span* followed the same trend and it was lowest for AZM MP and increased as RIF content increased. Likewise, VMD and the relative *span* values changed accordingly. VMD and NMD differed considerably indicating a tendency to aggregation of the MP during analysis (Fig. 2) as also demonstrated by SEM morphological observations that revealed the presence only of small particles with consistent size, confirming the attribution of the observed larger populations to MP aggregates (Fig. 2). The quantified %DC is reported in Table 2 as well as the actual ratios of AZM/RIF 1:1, 2:1 and 1:2 compared to theoretical ratios. For both drugs, the obtained %DC are in reasonable agreement with the theoretical with average biases between 2.5 and 17%.

**Table 1:** Size distribution parameters of the obtained spray-dried MP and commercial powders.

	NMD (µm) ± S.D.	Span	VMD (µm) ± S.D.	Span
AZM MP	1.8 ± 0.5	2.46	60.8 ± 6.8	1.15
RIF MP	2.8 ± 0.8	4.54	137.2 ± 10.5	2.51
AZM/RIF MP 1:1	2.9 ± 0.9	3.81	87.9 ± 5.7	1.32
AZM/RIF MP 2:1	2.4 ± 0.7	2.98	56.5 ± 5.3	1.19
AZM/RIF MP 1:2	4.8 ± 1.2	5.90	91.3 ± 10.2	3.04
RIF powder	10.9 ± 2.3	20.59	111.1 ± 12.8	3.31
AZM powder	10.3 ± 1.9	3.50	150.2 ± 15.4	4.55

Results are mean ± S.D. Broadness of size distribution (span) was calculated using equation 1.

**Table 2:** Drug content quantification in MP combinations and relative biases compared to theoretical values.

	Theoretical AZM/RIF ratio	Actual AZM/RIF ratio	RIF DC % ± S.D.	AZM DC % ± S.D.	Bias (%)	
					RIF	AZM
AZM/RIF MP 1:1	1	1.06	48.7 ± 1.0	51.3 ± 1.0	2.6	2.5
AZM/RIF MP 2:1	2	2.15	31.7 ± 1.1	68.3 ± 1.1	3.9	3.4
AZM/RIF MP 1:2	0.5	0.64	61.1 ± 1.4	39.9 ± 2.6	8.0	17.3

Drug content in each AZM/RIF formulation was determined by a HPLC method. Calibration for RIF and AZM was performed in the 0.1–0.55 mg/mL (r^2^ = 0.9997) and 1–4 mg/mL (r^2^ = 0.9994), respectively, λ_max_ = 210 nm. All measurements were performed in triplicate and results expressed as mean ± S.D. %DC was determined using Equation 2.

### Determination of MICs, MBCs and FICIs/FBCIs

Given the fine microparticle size, drug apparent solubility was increased enough to avoid the use of organic solvents to perform the assays (see Supplementary Fig. S1). The antibacterial activities of each pharmaceutical preparation, expressed as MIC and MBC, are summarized in Table 3. AZM/RIF MP combinations exhibited an enhanced antimicrobial effect compared to single molecules. Particularly MBCs, measured at 72 h when adequate growth of colonies was achieved, were at least four-fold and eight-fold lower than drugs alone for AZM and RIF, respectively. FBCI calculation measured 0.19 for 1:1, 0.31 for 2:1 and 0.25 for 1:2 AZM/RIF MP, all indicative of synergistic killing activity. Overall, the extracellular activity test did not highlight substantial differences among the three AZM/RIF MP combinations.

**Table 3:** Extracellular antimicrobial activity of the microparticle formulations against *R*. *equi* ATCC 33701.

	MIC (mg/L)	FICI	MBC (mg/L)	FBCI
AZM MP	0.5	—	8	—
RIF MP	0.125	—	16	—
AZM/RIF MP 1:1	0.06/0.06	0.6	1/1	0.19
AZM/RIF MP 2:1	0.125/0.06	0.73	2/1	0.31
AZM/RIF MP 1:2	0.03/0.06	0.54	1/2	0.25

The values of FICI and FBCI depend on the calculation model of Equation 3.

### Intracellular antibacterial activity

**Figure 3:**
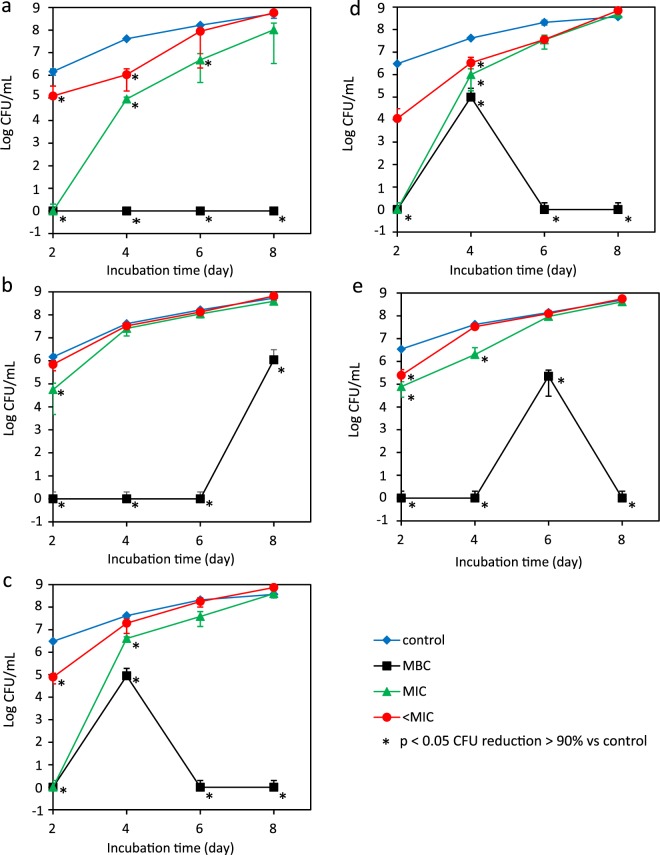
Colony count (log CFU/mL) at 2, 4, 6 and 8 days after treatment of infected THP-1 cells with AZM MP (**a**), RIF MP (**b**), AZM/RIF MP 1:1 (**c**), AZM/RIF MP 2:1 (**d**) and AZM/RIF MP 1:2 (**e**). Results are mean ± S.D. (some S.D. bars are smaller than the symbols). Comparison of different groups was performed by ANOVA with post-hoc Tukey HSD and Bonferroni, Kruskal-Wallis and Mann-Whitney tests. Friedman and Wilcoxon Signed Rank tests were carried out to compare readings at different incubation times. *p < 0.05 CFU reduction >90%.

More discriminating were the results of the intracellular activity assay in infected THP-1 cell line over time. The growth curves of *R*. *equi* ATCC 33701 in treated and untreated THP-1 cells are displayed in Fig. 3(a–e) showing the colony count. *R*. *equi* multiplied in the presence of THP-1 cells, increasing nearly up to log 9 CFU/mL within 8 days post infection. At day 2 after treatment, all tested drugs had an antimicrobial efficacy of >90% at MBC, MIC, and MIC/2, except for RIF at MIC/2 that showed a 52% CFU reduction compared to untreated controls (p < 0.05). At day 4, all concentrations of AZM MP and 2:1 AZM/RIF MP demonstrated an antimicrobial efficacy of >90% (p = 0.001). The same result was measured at the MBC and MIC of 1:1 and 1:2 AZM/RIF MP (p = 0.001) and at the MBC of RIF MP (p = 0.001). At day 6, such killing efficacy (>90%) was observed at the MBC of all drugs and the MIC of AZM MP (p = 0.012). Two-to-one AZM/RIF MP caused a CFU reduction of 83.5% and 82.8% at the MIC and MIC/2, respectively (p = 0.012), while 1:1 AZM/RIF MP had an intracellular efficacy of 81.6% at the MIC value (p = 0.012). Finally, at day 8, all combinations had a killing efficacy >90% at the respective MBCs (p = 0.012). Moreover, AZM MP at the MIC showed 80.5% CFU reduction (p = 0.013).

### Viability of BEAS-2B cell line after antimicrobial treatments

**Figure 4:**
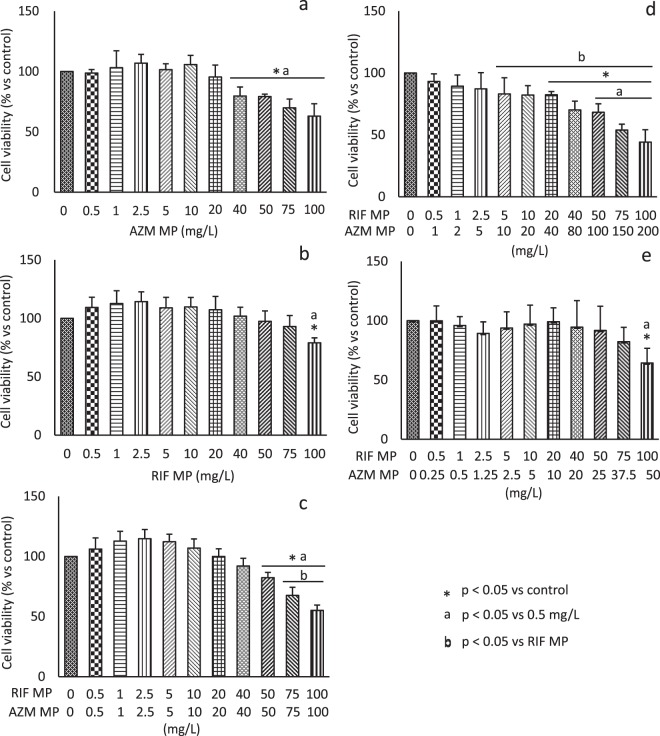
Viability of BEAS-2B cells after treatment with AZM MP (**a**), RIF MP (**b**), AZM/RIF MP 1:1 (**c**), 2:1 (**d**) and 1:2 (**e**). Data represent the mean ± S.D. Statistical analysis was performed by one-way and two-way ANOVA with post-hoc Tukey HSD and Bonferroni to determine statistical differences within and among groups. *p < 0.05 vs control; ^a^p < 0.05 vs 0.5 mg/L; ^b^p < 0.05 vs RIF MP.

**Figure 5:**
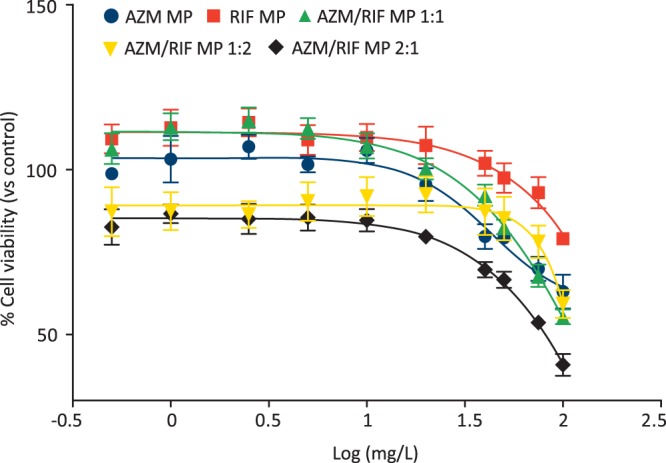
Measurement of IC_50_ values of AZM MP, RIF MP and their spray-dried combinations. IC_50_ was determined by automatic drawing of dose-response curves (mean ± S.D.). For AZM/RIF MP 1:1, 2:1 and 1:2, X-axis refers to RIF concentration in each combination.

As illustrated in Fig. 4, BEAS-2B cells exhibited a dose-dependent viability. A negligible cytotoxicity with a viability >90% was observed up to 20 and 75 mg/L of AZM MP and RIF MP, respectively. Such a result (viability >90%) was found up to 100 mg/L of AZM/RIF MP 1:1, 75 mg/L of AZM/RIF MP 1:2 and 3 mg/L of AZM/RIF MP 2:1, that maintained a cellular viability >80% up to 60 mg/L. ANOVA did not reveal significant statistical differences between AZM MP and the combinations as well as between RIF MP and AZM/RIF MP 1:2. Statistical significance was found at 75 and 100 mg/L/RIF between RIF MP and drug combination 1:1 (p ≤ 0.001) as well as at concentrations > 5 mg/L/RIF of AZM/RIF MP 2:1 (p ≤ 0.035). Figure 4a–c pinpoints statistical significances obtained by comparing in pairwise the negative control and the minimum tested concentration >MIC (0.5 mg/L) to higher doses. IC_50_ was ≥ 134.5 and 185.5 for AZM MP and RIF MP, respectively. Total IC_50_ of AZM/RIF MP 1:1, 2:1 and 1:2 was calculated ≥ 199.2, 239.1 and 307.1 mg/L, respectively (Fig. 5). According to toxicity categorization, all antimicrobial drugs demonstrated low toxicity. The CI values, obtained from Equation 4, of 1.2 (AZM/RIF MP 1:1), 1.6 (AZM/RIF MP 2:1) and 1.8 (AZM/RIF MP 1:2) indicated an antagonist outcome on cytotoxic effect of AZM/RIF combinations.

## Discussion

In clinical practice, the empirical approach towards the choice of antibiotics in a combination therapy is often insufficiently supported by *in vitro* and *in vivo* studies. In order to prevent the administration of potential antagonistic drugs and to identify differences in relation to killing activities of several antimicrobial combinations, *in vitro* assays represent the first necessary step for an insightful use of the combinatorial therapy, which is largely considered one of the most effective approach to contrast the emergence of antibiotic resistance and to enhance antimicrobial chemotherapy^31^.

This paper introduces a new potential approach to treat *R*. *equi* disease, employing AZM and RIF combined through co-spray-dried formulation method. Albeit AZM and RIF are administered routinely in combination, to the authors’ knowledge this is the first report of single formulations consisting of AZM plus RIF as microparticle dry powders, which might offer multiple therapeutic advantages. The improved dimensional and morphological features of the MP compared to commercial powders led to better dispersibility and higher solubility, especially for RIF in the combinations (Supplementary Fig. S1a–c), which are both critical parameters for achieving drug systemic and local therapeutic concentrations. Moreover, residual solvent in the spray-dried powders was between 1.3 and 2.6% w/w, low enough to not raise toxicity concerns (Supplementary Fig. S1d). Poor solubility of hydrophobic antimicrobial drugs, along with absorption and distribution issues, contribute to the well-known poor pharmacokinetic profile of antibiotics, which is responsible for the high treatment dosages as well as the documented toxicity^32^. In this regard, AZM has a very low bioavailability when administered orally to adult horses (1–7%) and humans (16–37%), while in foals it shows high variability (40 to 60%)^33–35^. Spray-dried MP combinations proved to disperse and dissolve more easily than single drug spray-dried MP and commercial powders, as displayed in Supplementary Fig. S1b. Therefore, taken alone, the promising features of the spray-dried combinations may help improving RIF and AZM bioavailability, reducing the required dosage and thus the known side effects.

The increased solubility allowed to avoid the use of organic solvents to perform *in vitro* assays as well, which is advantageous as low concentrations of organic solvents may alter the effect of antimicrobial drugs^36^, boosting or reducing the sensitivity of *R*. *equi* to the tested molecules^37^.

Although expected, the antimicrobial effect exerted by all drug combinations as compared to single molecules was remarkable. A partial AZM/RIF synergic effect seemed to occur, as suggested by the FICIs values, and the MICs of AZM/RIF MP combinations were halved compared to that of RIF MP, the most potent component. Our data seem to support the results from the, to the best of our knowledge, only screening work on the use of RIF/AZM combinations against *R*. *equi*, which described a synergic inhibitory median activity against several isolates^38^.

Strong support to the use of AZM/RIF combinations was provided by MBC determination. In fact, the concentration required for killing 99.9% of bacterial inoculum decreased significantly, clearly indicating a synergy. To determine MBC, CLSI recommended an incubation time of at least up to 48 h for gram-positive pathogens^18^; however, on the basis of our observations, the plates should be read at least up to 72 h to allow an adequate growth of *R*. *equi* colonies. Rifampicin showed a high MBC value when compared to the respective MIC. Such a finding was described previously^39^ and determined the synergic bactericidal activity of antibiotic combinations having MBCs at least eight-fold lower than RIF. Recently, it has been suggested the use of an effective bactericidal drug against rhodococcosis in order to avoid therapeutic failure due to bacteriostatic agents^40^. The synergic killing effect of AZM/RIF MP could enhance the outcome of medical treatment.

MIC and MBC values did not highlight marked differences in antibacterial activity among AZM/RIF MP combinations, which were in turn rather evident when testing the intracellular activity profiles over time. Indeed, long-term intracellular efficacy was correlated to a higher AZM concentration: the MIC of AZM MP showed an intracellular power of 80.5% until the last reading. The AZM/RIF MP 2:1 maintained a CFU reduction >80% at all tested concentrations up to the sixth day after treatment. This result suggests that granting this AZM/RIF ratio at the infection site could permit an extension of administration intervals and thus less frequent administrations. The AZM MP and AZM/RIF MP 2:1 had a comparable antibacterial effect showing CFU reduction >90% until the fourth day after treatment at all tested concentrations. However, MBC, MIC and MIC/2 of AZM/RIF MP 2:1 were at least four-fold lower than the corresponding values of AZM alone. By exploiting a higher MOI than that used in a previous research^41^, we were able to provide more restrictive conditions for testing the intracellular antibacterial efficacy and, consequently, more marked differences. However, the progressive and unexpectedly exponential growth of rhodococci over time observed in this study suggests the use of a lower starting MOI in future investigations.

The cellular viability after treatment with three fixed antimicrobial combinations demonstrated that toxicity was not boosted by combining AZM and RIF, as also proved by the measured IC_50_ values corresponding to low cytotoxic effect of the treatment with AZM and RIF. Additionally, the antagonist combinatorial effect resulting from Equation 4 may indicate lower harmful effects associated with combinations than single drugs. Recently, it has been found that AZM inhibits the release of pro-inflammatory cytokines, possesses an anti-apoptosis effect in bronchial epithelium and the ability to enhance post-injury reepithelization^42,43^. Therefore, AZM is deeply recommended for treating respiratory diseases^44^. Rifampicin is able to modulate certain transporters such as organic anion-transporting polypeptides (OATPs) and efflux carriers of the ATP-binding cassette (ABC)^45–47^. Therefore, it cannot be ruled out that the presence of RIF in combination may facilitate the accumulation of drugs inside cells and influence the epithelial response mechanism to drug challenge. These promising findings suggest that future research should be undertaken in this direction.

Moreover, the formulation of potentially inhalable powders opens new perspectives for the development of a technological platform for pulmonary treatment of *R*. *equi* infections. In this regard, it must be underlined that the proposed formulations are not prototypes of a potential final product, as further studies will be needed to optimize their properties granting delivery either through the oral or the pulmonary route. Although the pulmonary route is more attractive, the oral route cannot be excluded as no treatment is available for such combinations and therefore many aspects still need to be unraveled.

Overall, the current study suggests that a 2:1 AZM/RIF ratio at the target site may result in an extended duration of the effective antimicrobial activity and, consequently, in a prolongation of the administration interval with a lower required dosage.

Additionally, co-spray-dried AZM/RIF MP could be a clever and simple formulation strategy for facilitating the simultaneous administration of these anti-rhodococcosis first-line drugs. As anticipated above, future work will focus on exploiting spray-drying methods to produce this promising AZM/RIF association for which oral and, in particular, pulmonary routes of administration will be explored in the light of a potential higher compliance and efficacy with respect to current approaches. Furthermore, following pulmonary administration, the cited therapeutic effects of AZM in the respiratory tract could be enhanced while limiting its known side effects due to the high oral dosage and instability in the gastrointestinal tract^48^.

Finally, combining AZM and RIF is a well-known treatment strategy against several human bacterial infections, such as *Chlamydia* genus, gram positive cocci, non-tuberculous mycobacteria^49–52^ and, in our experience, equine pulmonary disease caused by *Streptococcus equi* subsp. *zooepidemicus*. Therefore, in the light of the observed performances of the proposed AZM/RIF MP combinations, the present work may serve as a stimulus for future research concerning their potential applications.

## Electronic supplementary material

Supplementary Figure S1

## Data Availability

All data generated or analysed during this study are included in this published article (and its Supplementary Information file).
