# Supplementary material for: In vitro performances of novel co-spray-dried azithromycin/rifampicin microparticles for Rhodococcus equi disease treatment
Source: Sci Rep. 2018 Aug 14;8:12149. doi: 10.1038/s41598-018-30715-z (PMC6092326; doi:10.1038/s41598-018-30715-z)
Supplement: Supplementary file 1 — Supplementary Figure S1 [file 41598_2018_30715_MOESM1_ESM.pdf]

***In vitro* performances of novel co-spray-dried azithromycin/rifampicin microparticles for *Rhodococcus equi* disease treatment**

Elisa Rampacci[1,2,*], Maria Luisa Marenzoni[1], Elisabetta Chiaradia[1], Fabrizio Passamonti[1], Maurizio Ricci[2], Marco Pepe[1], Mauro Coletti[1], Stefano Giovagnoli[2]

[1] Department of Veterinary Medicine, Centro di Studio del Cavallo Sportivo, University of Perugia, Via San Costanzo 4, Perugia, 06126, Italy

[2] Department of Pharmaceutical Sciences, University of Perugia, Via del Liceo 1, Perugia, 06123, Italy

* elisa.rampacci@gmail.com

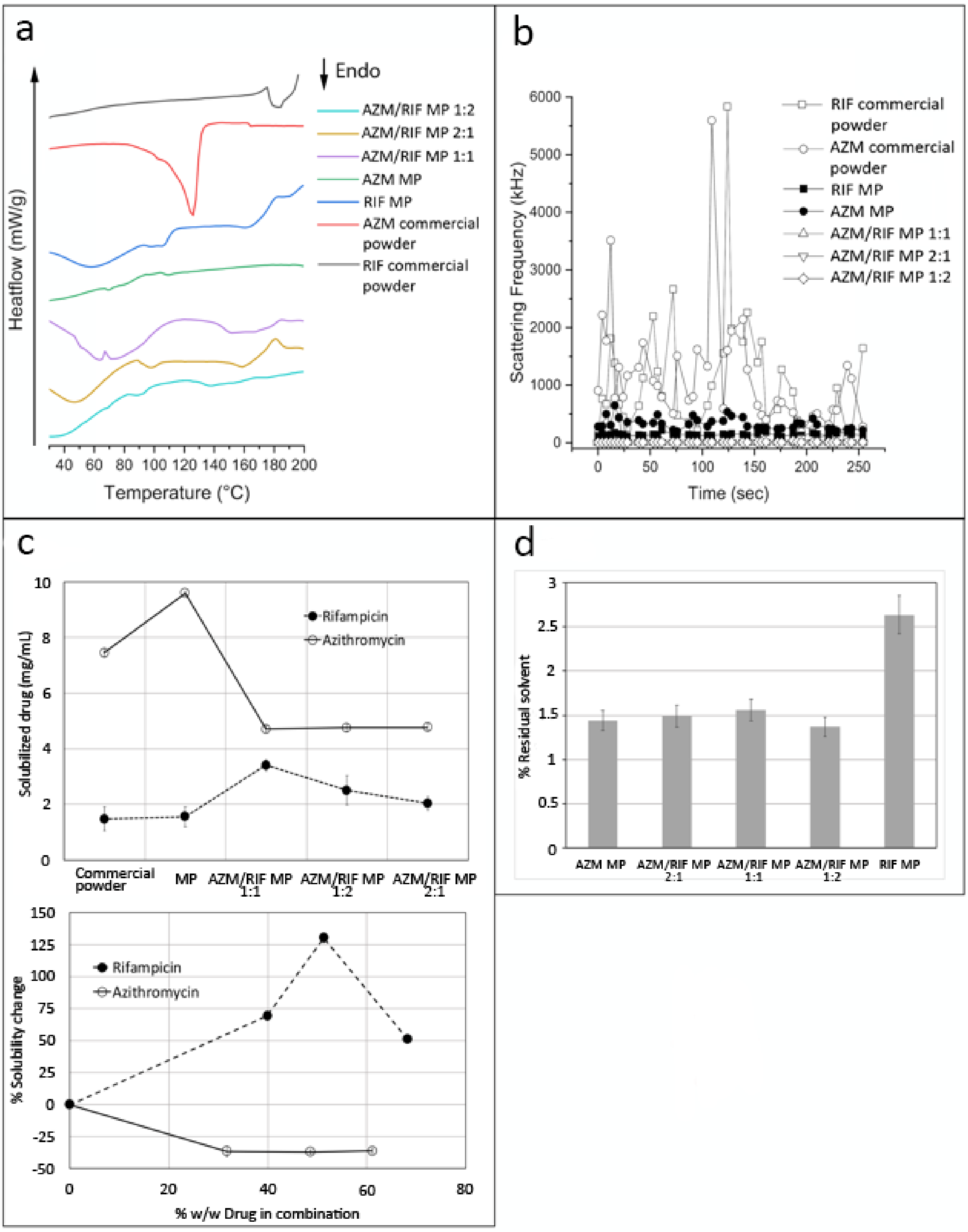

Supplementary Figure S1. a) Comparison of thermal profiles of AZM and RIF commercial powders and spray-dried MP of single drugs and combinations. Analyses were performed by differential scanning calorimetry in the 25-200 °C temperature range at 10°C/min heating rate. RIF commercial powder shows a characteristic melting/degradation pattern above 180°C, while AZM commercial

powder typical broad melting at around 110°C as a result of their crystalline nature. Spray-dried MP were amorphous as no melting was detectable, but only broad water bands below 100°C and typical RIF exothermic degradation at about 180°C.

b) Raw scattering signal of commercial powders and spray-dried MP. Measurements were performed by a scattering photocorrelator in dynamic mode at 20°C. Scattering signal is correlated with the presence of dispersed particles and the relative intensity is proportional to the size of the particles as well. Therefore, a higher scattering intensity is related to the presence of larger as well as more numerous dispersed particles. The signal intensity was in the order commercial powders >> single drug spray dried MP > spray dried MP combinations. In particular, scattering signal dropped nearly to zero for spray dried MP combinations. Therefore, two conclusions can be drawn: 1) spray-dried MP are characterized by much smaller dispersible particles and 2) these particles disperse and dissolve easily, especially when drug combinations are employed.

These results support a higher dispersibility and dissolution of spray-dried MP compared to commercial powders.

c) Solubility of commercial powders in comparison with spray-dried MP and combinations. The analysis was performed by using a HPLC method (refer to method section for details). The test was carried out by suspending proper amounts of the powders in 10 mM phosphate buffer pH 7.2 at 20 °C. After centrifugation the solution were diluted 1:2 in acetonitrile and submitted to analysis. AZM resulted the most soluble drug with values around 7.5 mg/mL while RIF showed a solubility in agreement with literature of 1.4 mg/mL. Spray-drying increased solubility of AZM alone and RIF in all the combinations in a way somehow influenced by AZM content. A solubility decrease was instead observed for AZM in the combinations compared to the drug alone. However, AZM solubility in the combinations remained at levels (4.5 mg/mL) granting complete dissolution of the powders

at the working concentrations used in this study. Overall, the combination of the two drugs was found to grant solubility of the powders compared to RIF alone.

d) Amount of residual solvent in the spray-dried powders. The amount of residual acetonitrile was determined by TGA as reported in the methods section. Levels were generally equal or below 1.5% w/w with the exception of RIF MP that showed a value reaching 2.6%. Overall, the concentration of residual solvent was well below the accepted limits thus not raising toxicity concerns.
